# Effects of Animal-Assisted Therapy on Gait Performance, Respiratory Function, and Psychological Variables in Patients Post-Stroke

**DOI:** 10.3390/ijerph18115818

**Published:** 2021-05-28

**Authors:** Ho-Jung An, Shin-Jun Park

**Affiliations:** 1 Department of Physical Therapy, Dongnam Health University, 50, Cheoncheon-ro 74 beon-gil, Jangan-gu, Suwon-si 16328, Gyeonggi-do, Korea; 0628jjang@hanmail.net; 2 Department of Physical Therapy, Suwon Women’s University, 1098, Juseok-ro, Bongdam-eup, Hwaseong-si 18333, Gyeonggi-do, Korea

**Keywords:** animal-assisted therapy, rehabilitation dog, gait training, rehabilitation motivation, stroke

## Abstract

Background: Animal-assisted therapy using dogs is being administered to patients post-stroke for the purpose of recovering psychological and physical activity. Objective: This study was conducted to confirm the effect of animal-assisted therapy using dogs on gait performance, pulmonary function, and psychological variables in patients post-stroke. All outcomes were analyzed using two-way repeated-measure analysis. Methods: In total, 30 post-stroke patients were divided into an experimental group (gait training by animal-assisted activity, *n =* 15) and a control group (gait training, *n =* 15). Gait performance (cadence, gait speed, stride length, symmetric index), respiratory pulmonary function (forced expiratory volume in 1 second (FEV1), forced vital capacity (FVC), peak expiratory flow (PEF), maximal inspiratory pressure (MIP), maximal expiratory pressure (MEP)), and psychological variables (rehabilitation motivation and depression assessment) were measured before and after eight weeks of intervention. Results: Gait performance, respiratory pulmonary function, and psychological variables significantly increased in the experimental group compared to the control group. Conclusion: Based on this study, it was found that animal-assisted therapy using dogs is an effective intervention for recovery of psychological and physical activity in patients post-stroke.

## 1. Introduction 

A stroke, caused by irreversible injury to the brain, is a chronic disease that can cause various difficulties in daily life due to the inability to use some parts of the physical body. Reduced physical activity post-stroke creates psychological and social difficulties [1,2,3]. The most typical psychological problem is depression, with a frequency of about 30% or more in patients post-stroke [4,5]. Depression has a negative effect on rehabilitation motivation, leading to missed rehabilitation periods and ongoing physical disabilities, as a result of which a vicious cycle of physical disorders is maintained [6,7]. According to previous studies, physical exercise has a positive effect on depression and rehabilitation motivation [8,9,10,11]. 

To achieve the optimal treatment outcome for patients post-stroke, neuro-physiotherapists must be made aware of the benefits of physical activity and the psychological and physical changes that it has on these patients. Physical activity requires interest, fun, and active participation. Physiotherapists should provide physical activity programs when the post-stroke patients are discharged and recommend other appropriate methods as needed. A resistance training program has been shown to improve the psychological variables, as well as the physical activity in post-stroke patients [12,13]. Among the various treatment methods available, animal-assisted therapy (AAT) can be used as a method for promoting patient physical activity [14]. 

AAT refers to the presentation of an animal to patients for the purpose of helping them recover, both psychologically and physically, as part of neurorehabilitation [14,15,16,17]. To this day, dogs are the primary animals utilized in AAT to improve independent activities of daily living and gait performance of patients post-stroke [18,19]. AAT with dogs can be combined with the training received in both physical and occupational therapy, which has been found to increase patient participation [20]. 

Gait training with a dog while wearing an adjustable elastic waist belt allows the dog to walk without disturbing the balance of the person. According to previous studies, the contraction of the trunk muscles induced by the waist belt affected intra-stability and induced postural control, resulting in better balance and gait performance of post-stroke patients [21,22]. Since the trunk muscles contain respiratory muscles, it has been observed that the stimulation during gait training with the adjustable elastic waist belt can improve the pulmonary function of patients post-stroke [23].

Gait training can also improve the pulmonary functions of post-stroke patients because the respiratory muscles are part of the trunk muscles, and these are stimulated when maintaining balance and gait [23]. 

Several studies have reported that AAT using dogs for therapeutic purposes can be used as an alternative therapy to treat neurological and psychiatric diseases [24] because it improves mental function [25], physical activity [26], cardiopulmonary pressures, and neurohormonal levels [27].

Although this dog training approach has been verified in various studies, AAT using dogs has never been reviewed as a randomized controlled trial (RCT) study design to identify changes in post-stroke patients’ psychological and physical activities. Therefore, the purpose of this study is to document the effects of AAT using dogs on gait performance, pulmonary function, rehabilitation motivation, and depression of patients post-stroke.

## 2. Methods

### 2.1. Design 

This study was conducted as an assessor-blinded RCT and approved by the institutional review board of Yong-in University: No. 2-1040966-AB-N-01-1902-HSR-130-1. This study was conducted in accordance with the ethical principles of the Helsinki Declaration. 

All participants were randomly divided into an experimental group receiving AAT gait training and a control group receiving the general gait training.

The experimental group engaged in AAT gait training for 30 min on weekends (Saturday or Sunday) for a period of eight weeks (1 session per week). The control group received general gait training for 30 min on the same schedule. All participants received neurodevelopmental treatment every weekday, including physical and occupational therapy.

The neurodevelopmental treatment (NDT) program was performed as follows. The physiotherapist performed core strength training and taught patients balance strategy training methods (if the dog suddenly walks fast while wearing the adjustable elastic waist belt, immediate trunk control is required). In occupational therapy, patients received hand strengthening exercises and reaching training (hand control is also required when feeding with the paretic hand in dogs). 

Informed consent was obtained from all participants before the study. Randomization was assigned 1:1 by generating computer random numbers (web site: Randomization.com) (Accessed on 2 March 2020). One occupational therapist, unrelated to this study, was assigned as an assessor. The assessor was blinded to group assignment, intervention effects, and data processing.

**Figure 1:**
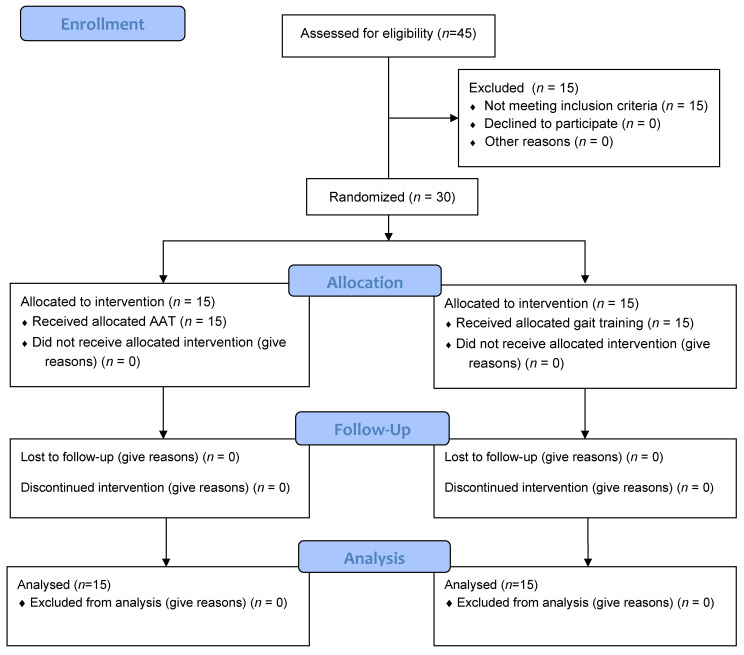
Flow diagram of participants.

The data were collected through assessment before and after eight weeks of intervention. None of the participants, including the pilot study, quit the study before the intervention was completed. The flow diagram of recruitment is depicted in Figure 1.

### 2.2. Sample Size Calculation 

Sample size was calculated using the G*Power software (G*Power 3.1.9.2, Heinrich-Heine-Universität, Düsseldorf, Germany). A pilot study was conducted on eight post-stroke patients. The effect size of 1.15 (cadence), 1.42 (gait speed), and 1.21 (stride length) were derived using the mean and standard deviation of gait performance outcomes. Based on the effect size, input of significance level was 0.05, power 0.80, and the total required sample size was 26, 18, and 24, respectively. In this study, 30 participants were selected considering the potential dropout rate and were randomly assigned to two groups (15 participants each in the experimental and control groups).

### 2.3. Participants and Recruitment

The study population consisted of post-stroke patients admitted to a rehabilitation hospital located in Gyeonggi-do Province, South Korea, from March to October 2020. A total of 45 patients post-stroke were enrolled. Of these, 30 patients satisfied the inclusion criteria. The inclusion criteria were as follows: (1) chronic stroke patients (more than six months), (2) had a score of higher than 23 on the Korean Mini Mental State Examination, (3) had normal hearing and visual function, (4) had a Berg balance score of 44 or higher, and (5) did not have restricted movement of the lips for pulmonary function test. However, participants who were afraid of dogs or had dog allergies were not included. 

### 2.4. Dogs and Handlers

An individual with more than nine years of physical therapy experience as a canine handler who has completed a dog training course at Animal Jobs Direct in the UK and is certified to perform AAT by the Korean Association of Animal Physical Therapy was selected. The rehabilitation dog was a three-year-old American cocker spaniel, systematically trained by a handler for the AAT. Therefore, both the dog and the handler were considered suitable for stroke rehabilitation through AAT. The rehabilitation dog was deemed safe for patients, because it had been screened for personality traits such as boldness, aggressiveness, sociability, and exploration. Additionally, this rehabilitation dog satisfied the criteria for both veterinary infectious diseases and vaccinations. In this study, food was given as a reward to motivate the rehabilitation dog.

#### 2.4.1. Animal-Assisted Therapy for Stroke Rehabilitation

Participants wore gait orthosis suitable to their capabilities, and the dog wore a chest strap. During gait training, patients wore an adjustable waist belt. The waist belt could be extended from 75 to 100 cm, so it was adjusted appropriately according to the waists of the participants. Prior to gait training, the chest straps worn by the dog and the waist belt worn by the patient were connected using elastic lead strap. The 140 cm-long lead strap was made of elastic band material. To prevent falls, an additional safety belt was worn on the waist belt.

The handler stood behind patients to assist and observe gait. During parts of the training, the handler asked the participants to pet or feed the rehabilitation dog. The dog was placed on the paretic side when feeding with both the paretic and the non-paretic hand. In the latter case, the food was fed using trunk twisting [20].

The 1–2 weeks of training aimed to create intimacy and interest in the rehabilitation dog; for example, learning the body language and the language that the dog responds to and basic etiquette training such as sitting, lying down, waiting, walking, rolling, and feeding were practiced. For 3–4 weeks, straight walking, figure of 8 walking, and free walking were performed with the rehabilitation dog as directly gait training periods. Outdoor gait practice was performed for 5–8 weeks. During outdoor gait practices, the participant went on a walk with the dog on the path next to the hospital. Resting time was freely taken within the given time, and for safety reasons, the patient and dog were always accompanied by the handler (Table 1).

#### 2.4.2. Gait Training for Control Group

Participants wore gait orthosis suitable for their needs and performed gait training without rehabilitation dog. Gait training of free walking was performed indoors.

### 2.5. Assessments

The primary outcome was gait performance. The secondary outcomes were pulmonary function, rehabilitation motivation, and depression.

#### 2.5.1. Gait Performance

Wearable sensor BTS G-Walk^®^ (BTS Bioengineering Corp., Quincy, MA, USA) was used to assess participants’ gait performance. G-walk is a wireless system that measures the spatiotemporal gait parameter by tri-axis accelerometer, magnetic sensor, and three-axis gyroscope (G-sensor). The G-sensor was placed in a holder within the belt for measurement, located on the fifth lumbar vertebrae. Participants walked a length of eight meters at a comfortable speed to provide parameters, including cadence, gait speed, stride length, and symmetric index. Data were transmitted wirelessly via Bluetooth to the BTS G-studio software (BTS Bioengineering Corp., Quincy, MA, USA).

#### 2.5.2. Respiratory Function Test

A spirometer (Pony FX, COSMED Inc., Rome, Italy) was used to assess the participants’ respiratory function. First, the pulmonary function was measured three times with forced vital capacity (FVC) methods [28]. For an objective assessment, the highest values of FVC and forced expiratory volume in the one second (FEV1) were selected. Here, the difference between the FVC values should be within 5% and 150 mL, and for the FEV1, it should also be within 150 mL. Additionally, to prevent air leakage, the mouthpiece was used, and maximal expiratory was encouraged for six seconds. If there was coughing or sharp peaks in the airflow–volume curve, the test was stopped. The measurement could be repeated up to, but not more than, eight times for each participant. Rest was performed for five minutes to maintain a stable state in the sitting position. The researcher verbally explained the procedure for the tests and conducted one trial. The procedure was to hold the maximal expiratory for six seconds in the maximal inspiratory state after biting the mouthpiece and holding the nose. The measured variables of the pulmonary function are FVC, FEV1, and peak expiratory flow (PEF). The same device was used to measure the respiratory pressure. Respiratory pressure was measured by using a mouthpiece and repeating the maximal inspiratory and expiratory. The maximum measured value obtained from three repeated measurements was used. The respiratory pressure measurement variable is the maximal inspiratory pressure (MIP) and maximal expiratory pressure (MEP).

#### 2.5.3. Rehabilitation Motivation Assessment

The Korean version of the stroke rehabilitation motivation scale (K-SRMS) was used to evaluate participants’ rehabilitation motivation [29,30]. This tool consists of a 24-item, five-point Likert scale (completely disagree = 1 to completely agree = 5). The higher the score, the higher the rehabilitation motivation level. Items 5 and 11 are reverse evaluation items. The seven subscales of K-SRMS were as follows: (1) extrinsic motivation introjection (items 1, 14, 20); (2) amotivation (items 2, 8, 15); (3) intrinsic motivation knowledge (items 3, 9, 16, 21); (4) extrinsic motivation regulation (items 4, 10, 17); (5) extrinsic motivation identification (items 5, 11, 22); (6) intrinsic motivation stimulation (items 6, 12, 18, 23); (7) intrinsic motivation accomplishment (items 7, 13, 19, 24). The K-SRMS can be used as a tool to objectify the rehabilitation motivation of patients post-stroke in clinical care and research and has good test–retest reliability and internal consistency [29,30].

#### 2.5.4. Depression Assessment

The Beck Depression Inventory-II (BDI-II) was used to evaluate participants’ characteristic attitudes and symptoms of depression [31]. The 21 items of BDI-II were as follows: (1) sadness, (2) pessimism, (3) past failure, (4) loss of pleasure, (5) guilty feelings, (6) punishment feelings, (7) self-dislike, (8) self-criticalness, (9) suicidal thoughts, (10) crying, (11) agitation, (12) loss of interest, (13) indecisiveness, (14) worthlessness, (15) loss of energy, (16) changes in sleeping patterns, (17) irritability, (18) changes in appetite, (19) concentration difficulty, (20) tiredness or fatigue, and (21) loss of interest in sex. The original version items 10,16,17,18, and 21 were removed, following which the scale was considered more appropriate for the depression assessment of post-stroke patients and can also reduce their burden [32]. Therefore, in this study, a 16-item inventory was used, with each item consisting of a scale from 0 to 3.

### 2.6. Statistical Analyses

IBM SPSS Ver. 21.0 (SPSS Inc., Chicago, IL, USA) was used for statistical analyses. The normality was assessed using the Shapiro–Wilks test. Prior to analysis, we observed homogeneity using the independent samples *t*-test and Chi-square before comparing between groups, as shown in Table 2. The treatment effect of analysis was measured using two-way repeated-measure analysis. The changes in variables between time (time) were within-subject factors, the differences of variables between group (time * groups) were between-subject factors. When significant differences were *t*-test were performed for post-hoc analysis. The statistical significance of *p* value < 0.05 was employed.

## 3. Results

### 3.1. General Characteristics

The results showed no significant differences in gender, paretic side, pathogenesis, disease duration, age, height, weight, K-MMSE scores, and Berg balance scale scores between the experimental and control groups, confirming homogeneity between the groups (Table 2).

### 3.2. Changes in Gait Performance

There were significant time and time * groups found for cadence (F = 4.458, *p =* 0.044), gait speed (F = 8.911, *p =* 0.006), stride length (F = 4.458, *p =* 0.044), and symmetric index (F = 5.501, *p =* 0.023). The two groups showed significant changes in the gait function variables after intervention (Table 3). The experimental group showed significantly greater increases in cadence, gait speed, stride length, and symmetric index than the control group (Table 4).

### 3.3. Changes in Respiratory Function

There were significant time and time * groups found for FEV1 (F = 4.614, *p =* 0.041), FVC (F = 4.809, *p =* 0.037), PEF (F = 4.560, *p =* 0.042), MIP (F = 4.444, *p =* 0.044), and MEP (F = 4.460, *p =* 0.044). The two groups showed significant changes in the respiratory function variables after intervention (Table 5). The experimental group showed significantly greater increases in FVC, FEV1, PEF, MIP, and MEP than the control group (Table 6).

### 3.4. Changes in Rehabilitation Motivation

There were significant time and time * groups found for K-SRMS total score (F = 6.730, *p =* 0.015). The experimental group showed significant changes in the K-SRMS total score after intervention (Table 7). The experimental group showed significantly greater increases in K-SRMS total score than the control group (Table 8).

### 3.5. Changes in Depression

There were significant time and time * groups found for BDI-II total score (F = 4.478, *p =* 0.043). The experimental group showed significant changes in the BDI-II total score after intervention (Table 9). The experimental group showed a significantly greater decrease in BDI-II total score than the control group (Table 10).

## 4. Discussion

This study found that AAT training with dogs to improve psychological and physical activity in post-stroke patients increased rehabilitation motivation, pulmonary function, and gait speed. Moreover, this study showed that it was more effective for all variables compared to the control group. This indicates the importance of AAT in nervous system rehabilitation. The strength of this study is that it is the first RCT study that confirms the effects of AAT on post-stroke patients. The effect of AAT with dogs is in line with previous cases, and pilot studies that found it to be effective in improving gait performance, gait speed, and rehabilitation participation [18,19].

The participants in this study completed a daily NDT program (core muscle, hip and ankle strategy, and gait training). This is a therapeutic exercise designed to prevent falls among post-stroke patients. Because core muscles, hip and ankle strategy, and gait are included when performing AAT, it can be incorporated into general physical therapy [20]. Participants who received AAT were able to perform it effectively because they followed the NDT program.

When compared with other AAT studies that applied to post-stroke patients, a smaller dog was used in this study. As populations become more urbanized, it will be important to understand the role of small dogs in AAT. In a previous study, a large dog was used as an alternative to canes [19]. Gait training using a big dog improves the symmetry of gait and the use of muscles, so it was found that there was a significant increase in gait speed than when a cane was used [19]. However, in the previous study, a walking aid used instead of the cane (a dog wearing a leather harness mounted with metal bars) can apply vertical pressure to the dog as the patient presses the metal bars during gait training [19]. This can give stress to the dog. The introduction of dogs into the treatment setting in the AAT has not yet raised great concern, but caution should be exercised, as there were behavioral signs of distress, and a rise in dogs’ cortisol levels were reported during the AAT [33]. To prevent this, participants in this study performed an AAT through a walk with dogs for eight weeks. AAT through a walk with dogs may prevent the dog from getting stressed [34].

In Korea, many people prefer to own small rather than large dogs [35]. As urbanization progresses, preference for small purebred dogs is higher in countries where conditions for raising large dogs are not available [35]. Bigger dog breeds are still preferred for AAT, but small-dog AAT is also a way to increase the treatments participation of the elderly [36]. Therefore, small-dog AAT can be used as an intervention method to improve the psychological and physical activity of post-stroke patients.

To improve gait speed, intra-stability such as core strength is an essential prerequisite. Previous studies have demonstrated the effectiveness of pelvic stability, for example, pelvic compression belts, for gait performance [22]. It is noteworthy that the dog harness used in this study is an adjustable elastic waist belt. The waist belt, which is not a hand-held type, frees the arms and is mainly used when jogging with a dog. In this study, since the dog can suddenly pull the patients, wearing a waist belt requires contraction of the trunk muscles to prevent disruption of the body balance from the sudden pulling force of the dog. It was possible to recognize trunk control during gait through attentional concentration. In the previous study, there was a strong correlation between gait, trunk motor control, and attentional concentration; therapeutic exercise through attention improved gait performance [37]. These results support our results. Therefore, it can be seen that AAT that utilizes trunk control while patients concentrate their attentional concentration during gait through an adjustable elastic waist belt is more efficient for gait performance than the typical gait training used in the control group.

The experimental group showed an increase in pulmonary function compared to the control group. Pulmonary function can be used to assess respiratory muscle strength. Usman et al. (2020) reported an increase in pulmonary function after treadmill training [38]; likewise, Alqahtani et al. (2020) reported an increase in pulmonary function with low-intensity aerobic training [23]. Since the respiratory muscles are part of the trunk muscles, it can be considered to improve pulmonary function by being stimulated by the trunk muscles during walking. According to this study, increased pulmonary function is attributed to gait training through AAT and trunk control increased by the waist belt during AAT.

Psychological variables such as depression and rehabilitation motivation both showed changes in the AAT group compared to the control group. AAT has a significant positive effect on patients’ general mood and wellbeing [18]. Post-stroke patients often have difficulty during rehabilitation due to negative emotions such as depression [6,7]. However, Theis et al. (2020) showed that AAT triggers positive emotions and low levels of arousal in patients with acquired brain injury [39]. Changes in mood after AAT can have an effect on depression and rehabilitation motivation due to the positive emotions generated in patients while enjoying spending time with the animal [40,41]. These findings are consistent with previous studies showing that AAT improves patients’ post-stroke memory [39], patient participation [20], and mood [18]. As a result, AAT can be seen as boosting rehabilitation motivation and decreasing depressive symptoms, because the therapeutic atmosphere is light, stress is low, and the activity itself (dog walking) incites pleasure.

Physiotherapists treating post-stroke patients should be aware of the existing benefits of therapeutic exercises and the importance of current available AAT for these patients. The physiotherapist can provide an enjoyable exercise program when the post-stroke patients are discharged from the rehabilitation environment. Addressing negative emotions such as depression and anxiety through dog-based AAT can enhance recovery for stroke patients and their families and increase rehabilitation motivation to start and maintain future exercises. Moreover, AAT can improve patients’ pulmonary function and gait performance. ATT using small dogs can be considered a valuable tool for rehabilitation of stroke patients.

Nevertheless, this study has several limitations. First, different types of dogs could be used for AAT, but the dog used in this study was limited to one breed: a trained American cocker spaniel. One species of dog was used because there was no method to employ a variety of dogs with matching gait speed. Second, during AAT, resting time was freely provided according to the patients’ fatigue levels. Thus, condition management cannot be generalized. Third, in this study, there are no objective evaluation data to correlate trunk muscle control of the waist belt with gait change when performing AAT. We indirectly identified changes in trunk muscle strength using MIP and MEP, which were evaluated in a static state. Another limitation is that questionnaire surveys such as depression and quality of life are inevitably affected by numerous extraneous variables such as varying daily conditions and recent eating habits. Due to these limitations, these results cannot be generalized to the post-stroke patient population. However, we believe that the study results suggest that walking with dogs may have a positive effect on the psychological and physical activity of post-stroke patients.

## 5. Conclusions

We observed changes in gait performance, pulmonary function, and psychological variables in stroke patients with AAT using a dog. Our findings indicate that AAT can be considered a rehabilitation method that improves depression and rehabilitation motivation and increases pulmonary function, respiratory muscle strength, and gait parameters.

## Figures and Tables

**Table 1:** Animal-assisted therapy for stroke rehabilitation.

Classification	Weeks	Time	Program
Early stage	1 week	30 min	Communication for patient/dog relationship
	2 weeks	30 min	
Middle stage	3 weeks	30 min	Gait training with dog indoors (straight walking, figure of 8 walking, and free walking)
	4 weeks	30 min	
Final stage	5–6 weeks	30 min	Gait training with dog outdoors (walk with a dog at the promenade)
	7–8 weeks	30 min	

**Table 2:** General characteristics of the participants.

Classification	Experimental Group (*n =* 15)	Control Group (*n =* 15)	X^2^/t	*p*
Gender (male/female)	9/6	9/6	0.001 ^b^	1.000 ^b^
Paretic side (right/left)	8/7	7/8	0.133 ^b^	1.000 ^b^
Pathogenesis (infarction/hemorrhages)	9/6	11/4	0.600 ^b^	0.700 ^b^
Disease duration (months) ^a^	12.93 ± 3.45	13.60 ± 3.64	0.515 ^c^	0.611 ^c^
Age (years) ^a^	60.93 ± 8.24	63.93 ± 7.68	1.032 ^c^	0.311 ^c^
Height (cm) ^a^	165.67 ± 9.39	164.47 ± 10.05	−0.338 ^c^	0.738 ^c^
Weight (kg) ^a^	70.60 ± 8.19	69.87 ± 6.96	−0.264 ^c^	0.794 ^c^
K-MMSE (score) ^a^	27.07 ± 1.39	26.60 ± 1.64	−0.842 ^c^	0.407 ^c^
BBS (score) ^a^	47.80 ± 2.76	47.07 ± 3.08	−0.687 ^c^	0.498 ^c^

^a^ Values are means ± SD, ^b^ Chi-square test between two intervention groups, ^c^ independent *t*-test between two intervention groups. Experimental group: gait training by AAT. Control group: gait training. K-MMSE: Korean Mini Mental State Examination. BBS: Berg balance scale.

**Table 3:** Changes in gait performance before and after intervention on two intervention groups.

Classification	Before Intervention ^a^	After Intervention ^a^	Within Group Change ^b^	%	Time		Paired-t	
					F	*p*	t	*p*
Cadence (steps/min)								
Experimental group	88.11 ± 12.11	106.18 ± 11.35	18.07 ± 2.05	20.30	80.145	0.001 **	8.833	0.001 **
Control group	86.13 ± 12.57	89.53 ± 13.88	3.40 ± 1.25	3.95			2.717	0.017 *
Gait speed (m/s)								
Experimental group	0.75 ± 0.11	1.04 ± 0.12	0.29 ± 0.02	38.67	168.846	0.001 **	14.645	0.001 **
Control group	0.72 ± 0.17	0.76 ± 0.18	0.04 ± 0.02	5.56			2.593	0.021 *
Stride length (m)								
Experimental group	1.03 ± 0.08	1.18 ± 0.08	0.15 ± 0.01	14.56	102.924	0.001 **	10.305	0.001 **
Control group	1.01 ± 0.15	1.02 ± 0.14	0.02 ± 0.01	0.99			2.229	0.043 *
Symmetric index (%)								
Experimental group	80.23 ± 5.66	85.42 ± 5.45	5.19 ± 1.04	6.47	37.313	0.001 **	4.984	0.001 **
Control group	77.10 ± 5.31	79.38 ± 6.00	2.28 ± 0.64	2.96			3.556	0.003 **

^a^ Values are means ± SD; ^b^ values are means ± SEM; * *p* < 0.05; ** *p* < 0.01. Experimental group: gait training by animal assisted therapy. Control group: gait training.

**Table 4:** Changes in gait performance between intervention on two intervention groups.

Classification	Between Group Change ^a^	Effect Size	Time * Groups		Independent-t		NNT (n)	ARR (%)
			F	*p*	t	*p*		
Cadence (steps/min)								
Experimental group	−14.67 (−19.59, −9.76)	2.23	4.458	0.044 *	−6.117	0.001 **	6	16.35
Control group								
Gait speed (m/s)								
Experimental group	−0.25 (−0.30, −0.19)	3.57	8.911	0.006 **	−9720	0.001 **	3	33.11
Control group								
Stride length (m)								
Experimental group	−0.13 (−0.17, −0.10)	2.60	4.458	0.044 *	−8.052	0.001 **	7	13.57
Control group								
Symmetric index (%)								
Experimental group	−2.91 (−5.44, −0.38)	0.87	5.501	0.023 *	−2.381	0.026 *	28	3.51
Control group								

^a^ Values are the mean (95% CI), * *p* < 0.05, ** *p* < 0.01. Experimental group: gait training by AAT. Control group: gait training. NNT: number needed to treat = 1/ARR. ARR: absolute risk reduction = EER (experimental event rate) − CER (control event rate).

**Table 5:** Changes in respiratory function before and after intervention on two intervention groups.

Classification	Before Intervention ^a^	After Intervention ^a^	Within Group Change ^b^	%	Time		Paired-t	
					F	*p*	t	*p*
FEV1 (ℓ)								
Experimental group	2.29 ± 0.45	2.90 ± 0.40	0.61 ± 0.04	26.64	117.676	0.001 **	16.380	0.001 **
Control group	2.18 ± 0.33	2.43 ± 0.37	0.25 ± 0.07	11.47			3.534	0.003 **
FVC (ℓ)								
Experimental group	2.58 ± 0.33	3.13 ± 0.31	0.55 ± 0.06	21.32	47.056	0.001 **	9.419	0.001 **
Control group	2.46 ± 0.35	2.74 ± 0.41	0.28 ± 0.10	11.38			2.630	0.020 *
PEF (ℓ/min)								
Experimental group	252.00 ± 41.40	328.47 ± 43.17	76.47 ± 10.40	30.35	51.871	0.001 **	7.351	0.001 **
Control group	237.87 ± 51.06	278.93 ± 49.80	41.07 ± 12.57	17.26			3.266	0.006 **
MIP (mmHg)								
Experimental group	41.27 ± 10.76	54.93 ± 9.05	13.67 ± 1.22	33.10	61.318	0.001 **	11.224	0.001 **
Control group	38.47 ± 10.18	43.47 ± 9.21	5.00 ± 2.05	13.00			2.440	0.029 *
MEP (mmHg)								
Experimental group	49.73 ± 10.78	69.80 ± 9.81	20.07 ± 1.35	40.36	67.329	0.001 **	14.821	0.001 **
Control group	47.87 ± 10.07	57.73 ± 10.60	9.87 ± 3.39	20.60			11.224	0.001 **

^a^ Values are means ± SD, ^b^ values are means ± SEM, * *p* < 0.05, ** *p* < 0.01. Experimental group: gait training by AAT. Control group: gait training. FEV1: forced expiratory volume in 1 s. FVC: forced vital capacity. PEF: peak expiratory flow. MIP: maximal inspiratory pressure. MEP: maximal expiratory pressure.

**Table 6:** Changes in respiratory function between intervention on two intervention groups.

Classification	Between Group Change ^a^	Effect Size	Time * Groups		Independent-t		NNT (n)	ARR (%)
			F	*p*	t	*p*		
FEV1 (ℓ)								
Experimental group	−0.36 (−0.53, −0.20)	1.64	4.614	0.041 *	−4.618	0.001 **	6	15.17
Control group								
FVC (ℓ)								
Experimental group	−0.27 (−0.52, −0.02)	0.82	4.809	0.037 *	−2.257	0.034 *	10	9.94
Control group								
PEF (ℓ/min)								
Experimental group	−35.40 (−68.83, −1.97)	0.79	4.560	0.042 *	−2.169	0.039 *	7	13.09
Control group								
MIP (mmHg)								
Experimental group	−8.67 (−13.55, −3.78)	1.33	4.444	0.044 *	−3.636	0.001 **	4	20.10
Control group								
MEP (mmHg)								
Experimental group	−10.20 (−17.67, −2.73)	1.02	4.460	0.044 *	−2.796	0.012 *	5	19.76
Control group								

^a^ Values are the mean (95% CI), * *p* < 0.05, ** *p* < 0.01. Experimental group: gait training by AAT. Control group: gait training. NNT: number needed to treat = 1/ARR. ARR: absolute risk reduction *=* EER − CER. FEV1: forced expiratory volume in 1 s. FVC: forced vital capacity. PEF: peak expiratory flow. MIP: maximal inspiratory pressure. MEP: maximal expiratory pressure.

**Table 7:** Changes in rehabilitation motivation before and after intervention on two intervention groups.

Classification	Before Intervention ^a^	After Intervention ^a^	Within Group Change ^b^	%	Time		Paired-t	
					F	*p*	t	*p*
K-SRMS (total score)								
Experimental group	78.73 ± 7.85	90.80 ± 5.00	12.07 ± 0.90	15.33	103.353	0.001 **	13.454	0.001 **
Control group	76.67 ± 7.79	78.73 ± 9.48	2.07 ± 1.06	2.69			1.946	0.072

^a^ Values are means ± SD, ^b^ values are means ± SEM, ** *p* < 0.01. Experimental group: gait training by AAT. Control group: gait training. K-SRMS: Korean version of the stroke rehabilitation motivation scale.

**Table 8:** Changes in rehabilitation motivation between intervention on two intervention groups.

Classification	Between Group Change ^a^	Effect Size	Time * Groups		Independent-t		NNT (n)	ARR (%)
			F	*p*	t	*p*		
K-SRMS (total score)								
Experimental group	−10.00 (−12.85, −7.15)	2.63	6.730	0.015 *	−7.193	0.001 **	7	12.64
Control group								

^a^ Values are the mean (95% CI), * *p* < 0.05, ** *p* < 0.01. Experimental group: gait training by AAT. Control group: gait training. NNT: number needed to treat = 1/ARR. ARR: absolute risk reduction *=* EER − CER. K-SRMS: Korean version of the stroke rehabilitation motivation scale.

**Table 9:** Changes in depression before and after intervention on two intervention groups.

Classification	before Intervention ^a^	after Intervention ^a^	within Group Change ^b^	%	Time		Paired-t	
					F	*p*	t	*p*
BDI-II (total score)								
Experimental group	14.47 ± 5.79	6.47 ± 3.18	−8.00 ± 0.89	−55.29	70.931	0.001 **	−8.998	0.001 **
Control group	15.00 ± 6.44	13.60 ± 4.76	−1.40 ± 0.67	−9.33			−2.075	0.057

^a^ Values are means ± SD, ^b^ values are means ± SEM, ** *p* < 0.01. Experimental group: gait training by AAT. Control group: gait training. BDI-II: Beck depression inventory-II.

**Table 10:** Changes in depression between intervention on two intervention groups.

Classification	Between Group Change ^a^	Effect Size	Time * Groups		Independent-t		NNT (n)	ARR (%)
			F	*p*	t	*p*		
BDI-II (total score)								
Experimental group	6.60 (4.31, 8.89)	2.16	4.478	0.043 *	5.913	0.001 **	2	45.96
Control group								

^a^ Values are the mean (95% CI), * *p* < 0.05, ** *p* < 0.01. Experimental group: gait training by AAT. Control group: gait training. NNT: number needed to treat = 1/ARR. ARR: absolute risk reduction = CER − EER. BDI-II: Beck depression inventory-II.

## Data Availability

Data generated and analyzed during this study are included in this article. Additional data are available from the corresponding author on request.
